# Supplementary material for: Are carbon emissions trading and green financial instruments synergistic? -Comprehensive quantitative research based on content analysis
Source: PLoS One. 2024 Mar 7;19(3):e0298601. doi: 10.1371/journal.pone.0298601 (PMC10919601; doi:10.1371/journal.pone.0298601)
Supplement: S1 Data — (ZIP) [file pone.0298601.s001.zip › Data/地级市Z_F工作报告.zip!/╣π╢1⁄2/╒┐╜¡╩╨/└┤╫╘╣1⁄2╓┌║┼í╛┬φ┐╦╩2╛▌═°í┐_2006.docx]

各位代表：

　　我代表市人民政府向大会作政府工作报告，请予审议，并请市政协委员和其他列席的同志提出意见。

　　2005年工作回顾

　　2005年是我市全面实现“十五”计划目标，各项工作取得新成就的一年。一年来，在中共湛江市委的正确领导下，我们以邓小平理论和“三个代表”重要思想为指导，深入贯彻党的十六大和胡锦涛总书记视察广东重要讲话精神，认真落实科学发展观，围绕建设现代化新兴港口工业城市和美丽的南方海滨城市的目标，积极实施“工业立市、以港兴市”发展战略，抓住机遇，发挥优势，团结实干，完成了市十一届人大三次会议确定的各项任务，全面实现了“十五”计划预期目标，在全面建设小康社会和现代化道路上迈出了新的步伐，为“十一五”的发展奠定了坚实基础。

　　综合经济实力再上新台阶。经济增速持续加快，经济总量不断壮大，财政收入显著增加，综合实力明显增强。全市生产总值657.81亿元，增长12.1%，净增106亿元，增速创近10年来新高。其中第一产业增加值138.3亿元，增长4.4%；第二产业增加值296.85亿元，增长15.3%；第三产业增加值222.66亿元，增长13.2%。海洋产业总产值412亿元，增长30.8%，居全省第3位。人均生产总值突破万元大关，达到10600元，增长11.9%。三次产业比例由上年的22.8∶41.7∶35.5调整为21∶45.1∶33.9。地方财政一般预算收入23.97亿元，增长21.3%；来源于湛江的财税总收入120.32亿元，增长32.6%。

　　工业发展活力和后劲增强。规模以上工业完成增加值216.18亿元，增长14.1%；完成总产值639.89亿元，增长16.8%；工业对生产总值增长的贡献率达44.6%，成为经济增长的主要动力。产业重型化趋势明显，轻重工业增加值比例为27.7∶72.3。工业企业经济效益综合指数255.6%，位列全省第一。东兴炼油500万吨扩建、包装材料尼龙薄膜、可口可乐和百事可乐等工业项目建成投产。东兴炼油厂成为年销售收入超百亿元的企业，中海油湛江分公司进入广东工业50强。实施名牌带动战略取得新成效，6个产品成为国家免检产品，1个产品被评为中国名牌产品，5个产品被评为广东省名牌产品，15件商标被评为广东省著名商标。工业园区引入珠三角产业转移项目52个，总投资44.37亿元。发展循环经济取得初步成效，发电、制糖、造纸、化工等行业废弃物循环利用率有较大提高，单位生产总值能耗比上年降低3.7%。成功举办’2005湛江工业博览会，签订投资合同额167.4亿元。

　　农业和农村经济取得新成效。农业结构不断优化，产业化水平提升，生产条件进一步改善。我市被评为全国农业产业化工作先进单位。完成省下达的粮食生产考核任务，糖蔗、香蕉、蔬菜、对虾、珍珠等农产品产量居全省第一。43家市级以上农业龙头企业销售收入81亿元，带动26万农户，户均增收2850元。大华糖业产业化经营模式被国家有关部门推广。农海产品有3个获认国家地理标志保护产品、10个获得国家绿色食品认证、10个被评为广东省名牌产品。水产品出口顺利通过欧盟和美国食品管理机构的实地检查。建成农产品检测中心。投资3.56亿元建设农田水利，投资4.88亿元实施城乡防灾减灾工程。完成造林24.4万亩。

　　县域经济和民营经济加快发展。县域完成生产总值277.31亿元，增长11.5%。各县（市、区）规模以上工业增加值增长均在10%以上；全部县（市）财政一般预算收入突破亿元。民营经济发展迅速，新增私营企业1207户，增长8%；新增个体工商户11458户，增长3.8%；民营经济增加值260.78亿元，增长14.7%，占全市生产总值近40%。

　　固定资产投资快速增长。全社会固定资产投资完成180.28亿元，增长17.9%，投资总量创历史新高。全年在建重点项目25个，实际完成投资53.35亿元，完成年度计划106.7%。珠三角成品油管道工程湛江段等7个项目竣工投产；奥里油电厂、德利化油器、珠江啤酒易地改造等续建和在建项目进展顺利。

　　基础设施和城市建设成效显著。湛江港发展实现大跨越，继30万吨级油码头建成后，25万吨级深水航道竣工通航，20万吨级铁矿石码头正式投产，港口“三大工程”全部完成，进一步增强了南方大港和大西南出海主通道的地位。渝湛高速公路粤境段建成通车，海湾大桥、疏港公路建设进展顺利，黎湛铁路河唇至湛江段增建二线动工建设。完成国道、省道和市通县（市）一级公路改造任务，建成县通镇公路258公里、镇通村委会公路硬底化1000公里、村通村公路4400公里，全市公路通车总里程达7800公里，公路密度每百平方公里62.5公里。城市规划和市政建设进一步加强，城市面貌发生可喜变化。城市总体规划、城镇体系规划和临港工业园区、华港工业小区控制性详细规划编制完成。积极推进土地储备，全年入库土地2161亩。建成寸金路延伸段和冯村生活垃圾处理场二期填埋区，湖港路、百蓬路、机场路延伸段和霞山污水处理厂建设顺利推进；市区旧城连片改造工程正式启动。建成渔港公园、绿塘河公园首期、灯塔公园、百乐园等4个公园和10多个小游园、小绿地，新增绿地52万平方米，荣获“国家园林城市”称号。廉江市被评为省林业生态县；徐闻珊瑚礁保护区升格为国家级自然保护区。投入新公交车100多辆，改善了市区公共交通条件。“百日环境综合整治行动”取得明显成效，市容环境有新改观。建成天然气储配站，市区开始规模化使用天然气。动工建设湛江电厂脱硫工程，建成后每年可减少二氧化硫排放5000吨。2005年国家环保总局公布全国环保重点城市环境综合质量考评结果，我市名列广东第一、全国第二。

　　第三产业优化提升。以港口货物贸易、运输等为重点的现代物流业快速发展。全市港口货物吞吐量6619.7万吨，增长29.9%，创造了一年净增1523.7万吨的历史纪录。其中，湛江港集团完成4098.4万吨，增长31.2%，增速比全国港口平均水平高11.2个百分点。公路、铁路货运量分别增长15.9%和24.1%。商贸服务业加快发展，消费需求持续畅旺。社会消费品零售总额269.25亿元，增长17.8%，增幅创近10年来新高。旅游、房产、汽车消费形成热点，标志着居民消费进入以住行为主的新阶段。邮政、电信业务总量52.76亿元，增长32.2%。专业市场日趋完善，粤西物流中心逐步形成，霞山水产品批发市场成为全国最大的对虾集散交易中心，荣获“全国十佳农产品批发市场”称号。全年接待游客560万人次，增长12.5%；旅游业总收入32亿元，增长12%。第三产业对经济增长的贡献率达39.9%。

　　外经贸工作平稳发展。外贸出口9.57亿美元，比上年略有增长。实际利用外资3896万美元，已有38个国家和地区客商在湛兴办实业或筹办项目。对外经济合作投资400万美元。

　　体制改革不断深化。初步建立国有资产监管体系框架，监管制度不断完善。实行产权制度改革的市属国有企业增加到95家，13家劣势国有企业有序退出市场。市属国有粮食企业改革基本完成，粮食市场宏观调控能力增强。公交车运营体制改革取得阶段性成效，湛江汽运集团通过兼并实现规模经营。规范国有产权交易行为，实施城区户外广告经营权公开拍卖。行政审批制度改革成效显著，清理和取消了一批行政审批和行政许可收费项目。投融资体制改革稳步推进，全市9家县（市、区）农村信用社联合社完成增资扩股任务。

　　社会事业全面进步。科技：新建3家省级工程中心，40%的大中型企业建立了技术开发机构；与中山大学建立联合实验室、检测中心和6个产学研合作基地；专利申请量和授权量分别增长17%和56%；高新技术产业产值100亿元，增长15%；科技进步对经济增长贡献率提高到46%。教育：投入1亿元实施中小学布局调整，新建学校11所，扩建学校155所，撤并学校182所；投入4000万元改造市区学校10所，增加学位3000个；投入4710万元完成157所老区学校改造任务；市二小、市二中新校区建成使用；普通高中发展迅速，优质学位明显增加。文化：市图书馆和少儿图书馆被评为国家一级图书馆；吴川市吴阳镇被评为全国历史文化名镇；雷州石狗、遂溪舞狮、吴川飘色、东海人龙舞、廉江舞鹰雄、雷州姑娘歌申报国家级“非物质文化遗产代表作名录”；湖光岩申报世界地质公园通过国家评审；成功举办国际舞狮邀请赛、首届红土文化艺术节。卫生：完成40间薄弱乡镇卫生院改造；农村人口参加新型合作医疗覆盖率35.44%；初步建立了突发公共卫生事件应急机制，有效预防和控制高致病性禽流感等疫情。体育：我市运动员在国际跳水大赛中获得7枚金牌；在世界帆板精英赛中获得冠军；在全国第十届运动会上获得3枚金牌，被省授予突出贡献奖。人口与计划生育圆满完成省下达的目标管理各项任务。人事部门人才引进和人才资源开发取得新成效。统计部门完成全国第一次经济普查工作任务。物价部门加强物价调控、监督和管理，维护市场价格秩序。档案部门提前完成农业、农村和社区档案工作任务。工商、质监、食品药品监督等部门在打假治劣、净化市场、保障群众健康等方面做了大量工作。外事、侨务、对台等部门积极加强与港澳台和海外华侨社团以及国外的经济文化交往。人防部门建成人防战备指挥所和现代通信指挥网络。海关、检验检疫、海事、边检、铁路等部门为全市经济发展作出新贡献。气象、地震、民族宗教、残联和地方志等工作取得新成绩。

　　人民生活水平稳步提高。城镇居民人均可支配收入9651.6元，增长8.2%；农民人均纯收入4250元，增长7.2%。全面免征农业税，农民负担进一步减轻。城镇、农村居民人均居住面积分别为27.7平方米和26平方米，均达全国平均水平。全市参加社会保险55.1万人，增长3%。新增就业岗位4.99万个，城镇登记失业率控制在3.2%以内。农村劳动力转移就业22.21万人。多渠道筹措资金，解决16万城乡群众最低生活保障和5.3万“五保户”供养问题。建成30宗农村饮水解困工程，解决了3万群众饮水困难。完成政府投资项目拖欠工程款清欠任务。改造乡镇敬老院27间，新建村级敬老院151间。改造农村贫困户危房7853户和水库移民危房3299户；市区建成廉租房8600平方米；发放住房公积金9730万元，支持干部职工购建住房，部分住房困难群众居住条件得到改善。居民储蓄存款余额505.7亿元，比年初增长12.2％。

　　民主法制和精神文明建设进一步加强。认真听取和采纳人大代表、政协委员的意见和建议，全年办理省、市人大代表议案及建议182件、市政协委员提案157件，满意率达98.4%。政务公开制度全面推进，市政府行政服务中心被授予“广东省人民满意的公务员集体”称号。加强行政监察和执法检查，开展价格、教育、医疗收费等纠风专项治理。加大审计执法力度，实施审计结果公告制度。积极开展平安湛江建设，社会治安综合治理工作首次跨入全省先进行列。保持打击走私高压态势，沿海56个乡镇建立反走私长效机制。认真处理群众来信来访，有效化解各种社会矛盾。全面完成省下达的安全生产控制指标。广泛开展“塑造湛江形象，争创文明城市”和以“四通五改六进村”为主要内容的创建生态文明村活动，14个单位被评为省级以上文明单位，4个社区被评为广东省文明社区，建设市级以上生态文明村342条，其中4条村被评为全国文明村、9条村被评为广东省文明村。

　　“十五”时期，是我市历史进程中不平凡的时期。全市经济加速发展，综合实力显著增强；城市面貌日新月异，社会各项事业全面进步；和谐湛江建设取得新成效，人民生活水平明显提高，实现了工业增加值、固定资产投资、港口吞吐量、外贸出口、财政收入“五个翻番”，人民生活实现了从温饱到小康的历史性跨越。“十五”计划任务的胜利完成，标志着我市经济和社会发展进入新阶段。

　　各位代表！这一切成绩的取得，是中共湛江市委正确领导的结果，是各级人民代表大会及其常委会和人民政协监督支持的结果，是中央、省驻湛单位和社会各方面支持的结果，是全市人民团结实干、艰苦奋斗的结果。在此，我代表市人民政府，向各级人大代表和政协委员，向在各个岗位上辛勤劳动、作出贡献的广大工人、农民、知识分子、干部、驻湛人民解放军、武警部队官兵、公安干警和社会各界人士，向所有关心和支持湛江建设发展的港澳台同胞、海外侨胞和国际友人，表示崇高的敬意和衷心的感谢！

　　在充分肯定成绩的同时，我们也清醒地看到，我市经济社会发展与落实科学发展观、建设和谐社会的要求还有差距，与全省经济社会发展水平、与人民群众的愿望和要求还有差距，还存在不少问题和困难：一是产业结构不够合理。临港重化工业产业关联度不强，产业链尚未形成，尤其是具有较高效益的下游产业薄弱；高新技术产业比重较低，现代服务业总体规模偏小，经济增长缺乏强有力的产业支撑。二是城乡一体化进程不快。中心镇建设缓慢，规模偏小，城镇发展的容纳能力不适应农村剩余劳动力加快转移的需要，农民增收途径不多，农村集体经济薄弱。三是经济外向度较低。外贸出口和利用外资总量偏小，与我市拥有的优势和地位不相称。四是城市规划建设管理与新时期发展的要求仍有差距。城中村、城郊结合部规划管理和改造明显滞后，城市管理存在明显的粗放特征，部分市区市容卫生管理不到位。五是社会事业仍未适应群众需求。文化教育卫生体育投入不足，社会保障能力不强，城镇就业压力较大。六是政府机关作风和行政效率未能适应加快发展的形势，投资环境特别是软环境有待进一步改善。少数部门一些领导干部大局观念、责任意识不强；一些工作人员办事拖拉，效率低下，工作时有失误。对这些问题，我们一定高度重视，认真对待，采取有效的措施，切实加以解决。

　　“十一五”时期的奋斗目标和2006年主要任务

　　“十一五”时期，是湛江加快建设现代化新兴港口工业城市和美丽的南方海滨城市、全面建设小康社会的重要时期。根据中共湛江市委八届五次会议的决定，市政府编制了《湛江市国民经济和社会发展第十一个五年规划纲要》（草案），提交本次大会审议。

　　“十一五”时期，湛江面临加快发展的良好机遇。我市拥有的港口、区位、资源、生态等优势，与国内外重化产业和资本转移，与国家实施能源发展战略，与西部大开发、泛珠三角区域合作以及中国—东盟自由贸易区的深入推进，与全省东西两翼振兴发展战略的实施等紧密地结合在一起，将有机会把潜在优势转化为经济优势，转化为现实生产力，实现后发式跨越发展。历史一再表明，机遇极为宝贵，稍纵即逝。抓住机遇，就会赢得一次跨越；错过机遇，就会贻误发展。全市上下必须牢固树立强烈的机遇意识，增强加快发展的紧迫感和责任感，充分发挥主观能动性，珍惜机遇，抢占先机，乘势而上，奋发有为，努力把机遇转化为发展的实际成果，实现经济社会发展的新跨越。

　　“十一五”时期我市经济社会发展的指导思想：坚持以邓小平理论和“三个代表”重要思想为指导，坚持以科学发展观统领经济社会发展全局，坚持以经济建设为中心不动摇，坚持以建成现代化新兴港口工业城市和美丽的南方海滨城市为总目标，坚持实施“工业立市、以港兴市”发展战略。加快振兴经济步伐，加快转变经济增长方式，加快推进改革开放，加快发展社会事业，加快建设社会主义新农村，加快提高人民生活水平。发挥后发优势，促进湛江科学发展，促进社会稳定和谐，为赶上全省率先基本实现社会主义现代化步伐、全面建设小康社会打下更为坚实基础。

　　“十一五”时期我市经济社会发展主要目标：2010年全市生产总值1200亿元，年均增长10%；农业增加值年均增长3%，工业增加值年均增长12.6%，第三产业增加值年均增长10.3%；三次产业比例优化为13∶51∶36；全社会固定资产投资年均增长12.5%；外贸出口总额年均增长10%，吸收外资年均增长10%；来源于湛江的财税总收入超200亿元，年均增长11%；港口货物吞吐量超1亿吨，年均增长11%；城镇居民人均可支配收入年均增长8%，农民人均纯收入年均增长6%；人口自然增长率控制在7‰以内；城镇从业人员社会保险参保率80%，农村人口参加新型合作医疗覆盖率85%以上；城镇登记失业率控制在3.8%以内；环境综合指标80分以上，城市人均公共绿地面积12平方米。争创全国卫生城市、国家环保模范城市、国家生态园林城市、全国林业生态城市和全国文明城市，继续保持“全国双拥模范城”的称号。

　　2006年是实施“十一五”规划的开局之年，是深入贯彻落实科学发展观、构建和谐社会，推动经济社会步入科学发展轨道的重要一年，也是站在新起点、实现新跨越、开创“十一五”发展新局面的重要一年。做好今年的工作，意义十分重大。

　　2006年我市经济和社会发展主要预期目标是：全市生产总值增长10%左右，其中第一产业增长3.5%，第二产业增长12%（工业增长12.5%），第三产业增长12.5%；全社会固定资产投资增长11%；外贸出口和实际吸收外资分别增长10%；地方财政一般预算收入增长11%左右；居民消费价格指数涨幅控制在3%左右；城镇登记失业率控制在3.5%以内；人口自然增长率控制在6.8‰以下；城镇居民人均可支配收入增长8%左右，农村居民人均纯收入增长6%左右。

　　为实现上述目标，今年必须着力做好以下工作：

　　一、强化工业主导地位，进一步提升工业发展水平

　　强力推进重点项目建设。全市安排重点建设项目39个，总投资410.11亿元，今年计划投资50亿元。健全各级领导联系重点项目责任制，明确完成时限；建立完善重点项目建设全过程主动服务制度，积极做好服务保障工作，及时解决建设中遇到的困难和问题。力争奥里油电厂、珠江啤酒易地改造、广东冠豪特种防伪纸等13个项目年内建成投产。加快东兴炼油厂原油适应性改造、50万吨中星石化重交沥青和98万立方米中油燃料油库二期工程等项目建设。积极推进10万吨蔗渣造纸项目前期工作，力促70万吨木浆项目早日动工。努力做好大型钢铁基地等重大项目争取工作。抓好《广东省工业九大产业发展规划》和《广东省东西两翼地区经济发展规划》中湛江投资项目的跟踪落实。

　　发展壮大支柱产业和骨干企业。引导生产要素向重大产业化项目和重点骨干企业集聚，发展壮大临港重化工业和农海产品加工两个重点产业，不断延伸和完善产业链条；着力培育壮大临港石化、能源、制糖、造纸、家用电器、羽绒、服装纺织等产业集群，加快重石化基地、电力基地和特色制造业基地建设。发展信息技术、生物、医药、新材料、新能源、环保等新兴产业项目，加快高新技术产业发展步伐。实行要素配置倾斜政策，培育发展一批主业突出、带动能力强、支撑作用大的大企业大集团。继续评选公布全市工业企业50强。

　　抓好工业发展载体建设。湛江经济技术开发区要大力提升产业层次和辐射带动功能，在推进科技进步和自主创新、培育先进产业等方面发挥示范带动作用；全力做好延伸区申报工作，力争年内获得国家批准。加强省级试验区和临港、官渡、麻章等工业园区的规划建设，做到当前发展需要与长远发展要求相结合，区分层次，合理布局，有序开发；完成东海岛试验区产业布局和开发建设规划。积极筹建省级高新技术产业开发区，推进省级农业科技园建设。年内争取一批项目在各类园区落户，一批项目建成投产，形成新的经济增长点。

　　以工业为主导壮大县域经济。从自身的特色和潜力出发，选择一批有资源优势、市场前景，对财政增收较好的工业项目加快建设，形成若干个支撑县域经济发展的支柱产业群。抓好与珠三角共建产业转移园区建设，完善基础设施和服务功能，吸引更多企业和项目进入，营造具有县域特色的工业产业基地。大力扶持具备一定规模、经营业绩良好、现有资产较佳的优势企业加快发展，壮大实力，使之成为投资扩张的主体和县域“块状经济”的龙头。

　　扶持发展中小企业和民营经济。加快社会信用体系建设，重点完善政府、银行、企业沟通合作制度，积极为中小企业提供信息、咨询、市场开拓和融资服务。扶持一批符合国家产业政策、具有行业竞争优势、发展前景良好的中小企业向小而专、小而特、小而精方向发展。全面落实《国务院关于鼓励支持和引导个体私营经济发展的若干意见》，清理限制性规定，打破行业垄断，完善金融服务，依法保护民营经济合法权益，营造平等竞争的法制环境、政策环境和市场环境，促进民营经济再上新台阶。

　　积极发展循环经济。加快编制全市发展循环经济的总体规划，确立相关目标、评价与核算指标和具体政策，建立循环工业促进机制，形成再生、增值、转化、降耗、环保的循环增长模式。积极在石化、造纸等企业推广利用余热、压差发电；在制糖企业推广利用蔗渣造纸、发电、制肥；在城区推广中水再利用，进一步提高工业循环用水率；广泛推行废品回收综合利用，建设资源再生产业基地，对固体废弃物进行无害化处理、资源化利用。启动循环经济示范园区建设。

　　二、统筹城乡发展，努力建设社会主义新农村

　　加快中心镇建设。全面落实省政府鼓励中心镇发展的各项政策，抓紧出台我市推进中心镇发展的政策措施。完善中心镇总体规划，进一步明确全市19个省级中心镇的发展重点、步骤、措施，加速建设。坚持“政府创新制度环境，市场活化经济资源，城乡统筹和谐发展”的总体思路，加快建设特色工业基地，增强聚集效应，引导乡镇企业向园区集中；加快推进农村集体建设用地使用权流转，引导土地向规模开发项目集中；加快市场、社区和文化、教育、卫生、体育等公共服务设施建设，引导农民向中心城镇集中，为农村剩余劳动力转移创造广阔空间，使中心镇成为连接城乡的节点和繁荣农村、服务农业、集聚农民的重要载体。

　　提升农业产业化水平。推进特色农业产业基地建设，重点发展优质粮食种植基地和“双高”糖蔗、北运菜、热带水果、城郊花卉、桉树速生丰产林生产基地；加快建设水产品和畜产品出口基地；以粤台农业（湛江）合作试验区和热带现代农业园区为载体，建设全省热带农产品基地；支持建设大型深水网箱养殖基地；推进林浆纸一体化基地建设，延长林业产业链；鼓励优质畜禽规模化饲养，积极发展节粮型畜牧业。主动调整农业发展模式，大力发展农村二、三产业，增加农民的非农收入。开展农产品和水产品保鲜包装、综合利用技术攻关，加快发展农产品加工业。改造提升和建设一批辐射范围广且能引导价格形成的区域性大型农产品批发交易市场，加快发展农产品现代流通业。发展农民专业合作经济组织，拓宽农民和农村集体经济增收渠道。支持市级以上农业龙头企业与广大农户之间建立利益共享、风险共担的联结机制，力争3年内带动全市三分之一以上的农户增加收入。加快建设现代渔港经济区，推动海洋资源综合开发。

　　着力改善农业生产条件。调整各级财政支出结构和国民收入再分配制度，逐步建立稳定的农业投入增长机制。积极实施国家农业综合开发项目，大力推广良种繁育、现代集约化设施种植养殖等先进适用农科技术，推进农业机械化，扶持农民购置先进适用农机具，提高农业科技水平。完成徐闻大水桥水库除险加固，推进20宗城市防洪、雷州青年运河灌区节水改造和雷州半岛扩大治旱工程建设。发动群众兴修水利设施，整治水渠2300条。完成县通镇公路改造172公里、镇通村委会公路硬底化800公里，新建改建桥梁30座。完成造林24万亩。加快农村电网改造，确保农用电供给。

　　扎实推进社会主义新农村建设。按照中央提出的“生产发展、生活宽裕、乡风文明、村容整洁、管理民主”的要求，大力推广徐闻、吴川、廉江等地生态文明村建设经验，掀起社会主义新农村建设热潮。各村要制定一个合理规划，发展一个特色产品，推进一批改造工程，建设一个公共村场，营造一个洁净环境，种上一片成荫绿树，动员和组织农民建设文明富裕安康的新生活。认真抓好农村村务公开和民主管理工作，为农村稳定和发展创造良好环境。加快实施农村安居工程，改造水库移民新村64条，努力推进农村贫困户危房和茅草房改造。完成自然村“村村通”广播电视工程。

　　三、加强城市规划建设管理，营造良好人居和创业环境

　　强化城市规划的龙头作用。完成城市总体规划和城镇体系规划的报批。组织编制市域资源环境保护规划和城市控制性详细规划，形成完整的规划体系。编制实施《湛江市城市近期建设规划》，明确“十一五”期间城市发展目标、建设重点和实施手段，加强对城市各项具体开发建设行为的统筹与调控，重点加强城乡结合部、城中村和市区人行系统的改造规划。应用系统理念规划城市配套设施，合理布局公共停车场、港湾式公交车站、居民活动场所等，完善和提升城市功能。严格控制国道、省道等交通主干线两侧建设，防止沿公路两旁线状布局工商业项目。依托即将建成的海湾大桥拓展港湾东岸，高起点、高标准规划建设海东新区，形成“一湾两岸”的城市发展格局。修订《湛江市城市规划管理办法》和制定《湛江市城市规划管理技术标准》，加强对规划工作的公开监督，规范规划修编和实施程序。用铁的手腕严格实施规划，坚决制止和查处违法建设行为。

　　推进基础设施和市政建设。实施湛江港30万吨级航道浚深工程，建设湛江港宝满港区集装箱码头一期、大型散装化肥、散货以及成品油专业码头，加快霞山、调顺、霞海港区技术改造，推进海安、流沙港和国家级硇洲中心渔港建设，逐步建立层次分明、结构合理、功能完善的港口体系。建成疏港大道，动工建设海湾大桥西连接线公路、市区至雷州一级公路和5个公路主枢纽站及8个县、镇级枢纽客运站场，大力支持黎湛铁路河唇至湛江段增建二线加快建设，推进湛徐高速公路、茂湛铁路和湛江铁路枢纽项目前期工作。完善城乡电网基础设施，确保500千伏港城输变电和奥里油电厂送电及其配套工程建成投产。加快构建城市基础设施体系，确保海湾大桥年内建成通车，动工建设和改建奋勇大道、南方路片路网首期、体育南路、北站路、站前路及城市出入口道路，续建百蓬路、机场路延伸段、湖港路和东盛路，启动湛江大道建设前期工作，改造人民南、文明路等5条道路路灯，加强市区无障碍公共设施建设，鼓励市辖区改造内街小巷。

　　加强生态环境建设。启动创建国家卫生城市、国家环保模范城市活动，推进环境友好型社会建设。以“生产发展、生活富裕、生态良好”为目标，推进国家级生态示范区、林业生态县和省级生态示范村镇创建工作。全面实施碧水、蓝天、宁静、洁净、生态和治污保洁工程，加强九洲江、鉴江、南渡河等重点流域和天然水体、农村水网的保护，加强地下水资源的保护和利用；继续实施赤坎江、绿塘河、文保河、霞湖片等排水截污整治工程；建成金沙湾观海长廊二期和霞山污水处理厂一期工程，启动建设赤坎污水处理厂二期管网工程和麻章污水处理厂；建设市区生活垃圾处理场一期封场工程，继续改造一批垃圾转运站和公厕；完善中心镇垃圾处理设施。严禁生产经营粘土实心砖。落实污染物排放总量控制目标，大力推广使用清洁能源，逐步实施餐饮业和汽车油改气工程。继续优化城市人居环境，完成霞湖公园改造，启动建设南国热带花园、滨湖公园、中澳友谊公园、绿塘河公园二期工程，年内建成10个以上小游园、小绿地，市区新增绿地面积100万平方米。

　　推进旧城区和城中村改造。坚持规划先行、整体开发、逐步推进、完善功能配套的原则，加快实施旧城区改造步伐，确保霞山汉口路片旧城改造取得实质性进展。采取“政府主导，市场化运作”的模式，加快城中村、城郊村改造进程，出台实施专项规章，坚决遏制违法建筑蔓延的势头，确保不再出现新的违法建筑，已有的违法建筑限期依法拆除。在城市总体规划指导下，采取“一村一策、一村一案”的方式，因地制宜，突出特色，努力把城中村和城郊村改造成为符合城市发展要求的现代新型社区、生态园林区和特色文化区，年内分别在霞山、赤坎和湛江经济技术开发区选村试点，精心组织实施。

　　进一步强化城市管理。坚持建管并重，规范管理，依法治城。完善“二级政府、三级管理、四级网络”的城市管理体制，试行管理重心下移和事权下放，充分发挥区、街道、社区在城市管理中的主体作用，发动群众积极参与城市管理。以市场化为取向，推进市容卫生、园林绿化、公共交通等管理体制和运营机制的创新。强化以街道为单位的市容环境卫生管理，制定实施《卫生街区标准》，全面开展创建卫生街区活动。进一步提高城市管理综合执法效能，重点加强市区出入口、次干道和内街小巷的综合治理，突出整治乱摆卖、乱张贴、乱拉挂、乱停放、乱堆挖和乱搭建行为，维护良好的市容市貌。建立城市管理热线投诉系统，及时解决市民反映强烈的问题。

　　四、深化重点领域改革，增创体制新优势

　　深化国有企业改革。健全以财务监管为核心的国有资产监管体系，加快委派财务总监、监事会进驻直管企业，实施《企业国有资产监督管理暂行办法》，完善国有资产收益收缴制度。整体规划、分类推进市属153家国有企业改革改制，完善法人治理结构，加快经营机制转换，盘活存量资产。建立完善资本市场服务体系，推动优势企业股份制改造，实现产权多元化。加快推进配套改革，促使国有劣势企业有序退出市场。全面完成国有粮食企业改革任务。

　　深化投资体制改革。实行投资项目核准制和备案制。进一步放开市场准入，推出一批项目面向市场招标，引入社会投资主体参与基础设施、社会事业和经营性公用事业建设。规范政府投资行为，充分发挥财政资金的引导和带动作用，广泛吸引民间投资和境外投资。

　　深化财政金融体制改革。加强财源建设，加大税费征管力度，实现财政收入稳步增长。完善部门预算、国库集中收付、政府采购和“收支两条线”制度，将国库集中支付改革的范围延伸到所有市直差额拨款事业单位。建立财政支出绩效评价体系，加大财政支出全过程监督，提高财政资金的使用效益。改革财政资金审批制度，全面推行财政扶持性项目公开招投标，提高财政资金投向的透明度。理顺市对区的财政收入分配关系，推广村财镇管的财会管理模式。构建良好的金融生态环境，大力推进市商业银行和农村信用社改革，促进地方金融事业发展。

　　深化城乡体制改革。加快建立以工补农、城乡互动、协调发展的新型城乡关系，促进城市基础设施向农村延伸，城市社会服务向农村覆盖，城市文明向农村辐射，加快城乡一体化进程，逐步缩小城乡差距。以消除城乡二元结构为目标，全面推进户籍管理、土地流转、劳动就业、社会保障等城乡配套改革和农村综合改革。积极开展以股份合作制为主要形式的农村集体资产管理体制改革，探索发展壮大村级集体经济的新路子。

　　深化事业单位改革。按照“统筹规划、突出重点、分类指导、稳步推进”的思路，加快事业单位结构调整。进一步规范事业单位机构性质和经费形式，加强机构编制管理，做好财政供养人员总量控制。深化事业单位人事制度改革，完善全员聘用合同制和岗位管理制度，新增人员全部公开招聘。推进生产经营性事业单位改企、改制，建立适应市场经济要求的运营机制。

　　五、扩大对外开放，推进招商引资

　　强化招商引资工作。抓住国际产业和资本向沿海地区转移的有利时机，坚持引进外资与产业结构调整优化相结合，引导外资投向重化工业、农海产品加工、高新技术产业和现代服务业、基础设施等领域，全力以赴主攻引进辐射力强、产业链长的大项目，引进龙头型、基地型大企业，争取更多世界500强企业、跨国公司和地区总部落户湛江。创新招商引资方式，扩大网上招商，建立对外推介项目库，有针对性地策划、包装一批可操作性强的项目推出招商。注重发挥我市海外华侨华人作用，拓展招商引资渠道。完善招商引资责任制，加大奖励、考核力度，充分调动各级招商引资的积极性。今年每个县（市、区）实际利用外资1000万美元以上。

　　努力保持出口稳步增长。主动适应出口退税新政策，调整企业出口策略。坚持科技兴贸、以质取胜，鼓励扩大一般贸易，加快发展加工贸易，注重提高出口产品质量和附加值。着力优化出口产品结构，培育优势品牌，扩大机电产品、高新技术产品和农海产品出口，不断开拓新的出口市场。积极应对反倾销、反补贴保障措施和其他国外贸易壁垒，提高抵御国际贸易市场风险能力。加快“大通关”建设，完善口岸配套设施，全面提升口岸竞争力。注重培育发展县域特色产业、特色商品出口基地。每个县（市、区）今年完成外贸出口3000万美元以上。

　　加强区域经济合作。充分利用中国—东盟自由贸易区、内地与港澳更紧密经贸关系安排以及泛珠三角区域合作平台，努力扩大与东盟、港澳台以及环北部湾、大西南等地区的交流和经贸合作，积极主动接受珠三角的产业转移。注重选择互补性强的国内外城市缔结为友好城市，提升与友好城市间的合作水平。实施“走出去”战略，鼓励有实力的企业到境外投资。办好2006年湛江工业博览会和湛江对虾节。

　　六、提升现代服务业水平，加快发展第三产业

　　发展港口物流业。充分发挥大西南出海主通道的优势和作用，精心打造港口服务品牌，加快培育建设石油、矿石、粮食、化肥等大宗货物集散中转基地；拓展港口腹地，深化与中西部地区货物进出口的中转合作，共同发展港口运输、货代、船代、仓储、货运场等相关项目；引入国内外有实力的船务公司和货代、船代企业来湛投资合作，拓展国际集装箱运输和中转、加工、仓储、贸易业务。力争全市港口货物吞吐量突破7500万吨。

　　发展商贸服务业。组织实施《湛江市商业网点规划》，改造提升一批重点商业圈，加快商贸物流、高档写字楼、购物中心等建设，加强与珠三角和港澳台服务业的合作，吸引国内外知名商家进驻建设大型超市和高档宾馆酒店，着力培育城市中央商务区。大力发展连锁经营、物流配送、电子商务等新型业态和现代流通方式，促进发展文化、中介、社区、公共服务等新兴服务业，逐步改造城区肉菜市场。扶持发展商业龙头企业，增强带动作用。以市场化运作为手段，加快培育发展会展业。推进信息化进程，加快信息资源的开发，普及信息技术的应用，发展广播电视、电信和电子信息服务业；加强政府网站建设，充分利用信息化手段提高公共服务水平。推动“万村千乡”市场工程建设，开拓农村消费市场，扩大服务业发展领域。

　　发展特色旅游业。加快旅游资源整合和集中统一开发，推动景区景点上档升级。逐步开发港湾及滨海体育休闲旅游、生态农业旅游、临港工业旅游和特呈岛、东海岛、硇洲岛等海岛旅游项目；开发半岛火山遗迹等新兴景点，建设环湖光岩自然生态旅游区；充分利用醒狮、飘色、人龙舞等红土文化艺术资源，进一步丰富旅游内容；举办湛江旅游美食节；建设旅游购物一条街，开发特色旅游商品，增强游客市场的吸引力。加强珠三角、港澳地区、大西南和粤桂九市等区域旅游合作，扩大旅游招商，拓展旅游客源市场。认真做好迎接“中国优秀旅游城市”复检和湖光岩风景区申报世界地质公园工作。

　　发展房地产业。进一步完善土地收购储备制度，完备用地手续，规范经营性房地产项目土地使用权出让行为，盘活存量建设用地，提高土地资源利用率和效益；政府出让土地坚持先修路、绿化、建配套设施，带动土地升值，再推向市场，提高土地公共收益。加快发展房地产业，实行小区连片开发，完善公共配套设施和物业管理。继续深化住房制度改革，全面推进住房公积金和住房货币分配制度。进一步激活和规范房地产市场，大力发展建筑业和装饰业。

　　七、建设节约型社会，协调发展社会事业

　　推进节约型社会建设。把节约理念贯穿于生产、流通、消费和社会生活各个领域，引导全社会形成“节约光荣、浪费可耻”的社会风尚。大力发展节能、降耗、减污、增效的产业和项目，广泛推广节能节水节材新工艺、新技术、新材料、新产品，力争年内单位生产总值能耗降低3%以上。深入开展建筑节能节材活动，城市照明推广采用节能灯具及节电自控设备，广泛应用农业喷灌、滴灌等技术，积极实施节水工程。突出抓好高耗能、高耗水等重点行业的100家重点企业的节能节水工作。在行政机关、事业单位倡导厉行节约，努力建设节约型机关、节约型单位。

　　增强自主创新能力。致力于建设创新型湛江，加快建设以企业为主体、市场为导向、产学研相结合的自主创新体系。以提高重点产业核心竞争力为重点，加强科技项目的实施工作，努力实现从跟踪模仿向创立自主品牌转变，从单项创新向系统集成创新转变，从各自科技攻关向联合攻关转变。继续实施名牌带动战略，争创更多国家、省名牌产品。争取年内组建各级工程中心6家、企业技术开发机构5家，培育特色专业镇5个。巩固和扩大校市合作，建立一批产学研示范基地。大力推进专利技术和成果转化示范工程，年内专利技术申请量和授权量分别增长10%以上。全市高新技术产业产值增长15%。

　　加快发展教育事业。加大基础教育投入，优化城乡校网布局。大力发展高中阶段教育，加快示范性高中建设。积极推进中小学布局调整和学校改造，继续扩大中学学位。实施农村义务教育学校学生生活设施和中小学危房改造工程，努力改善办学条件。认真落实农村义务教育免收学杂费政策，规范教育收费，努力解决群众上学难、上学贵问题。整合资源，推进校企联合办学，加快发展职业教育，支持驻湛高校做大做强。

　　努力建设文化大市。加快文化体制改革，逐步形成以公有制为主体、多种所有制共同发展的文化产业格局和统一、开放、竞争、有序的现代文化市场体系。建设一批民间艺术之乡，抓好中国民族民间文化保护试点和国家级“非物质文化遗产代表作名录”的普查及申报工作，推进南疆文化走廊建设。加强社区、企业、校园等文化建设，因地制宜深入开展农村特色文化村、文化室、文化节活动，丰富城乡群众精神文化生活。建成市广播电视中心。实施市区数字电视部分转换。建设市电子文档数据中心。开展第二届地方志编修工作。加强文艺人才引进和培养，壮大文艺队伍。

　　促进医疗卫生和体育事业发展。进一步完善疾病预防控制体系和公共卫生应急反应机制，做好高致病性禽流感、艾滋病等重大传染病的防控工作。强化医疗收费管理和整治，倡导建立平价医院、平价门诊、平价病房、平价药房，积极推行解决群众看病难、看病贵的办法。发展社区和农村卫生事业，重点改造37间乡镇薄弱卫生院，开展卫生村创建活动。完善新型农村合作医疗制度，年内实现农村人口参加合作医疗覆盖率达到60%以上。加快发展中医药事业。加强群众性体育设施建设，认真备战第12届省运会，办好市第七届体育节。

　　加强人口与计划生育工作。认真贯彻人口与计生工作法律法规，落实计划生育目标管理和层级动态管理责任制，抓好计生新机制试点，严格执行“一票否决”制度，优化计生技术服务，积极开展无政策外多孩生育、村（居）无政策外出生和优质服务年活动，加强流动人口计生管理和服务，综合治理出生人口性别比失衡问题，确保完成人口与计划生育工作各项指标任务。

　　全面做好各项社会工作。加强民兵、预备役部队和人民防空事业建设，继续抓好双拥优抚安置工作，巩固军政军民团结，争取第三次被评为“全国双拥模范城”。抓好民族宗教工作，全面落实侨务政策，依法保护归侨、侨眷合法权益。加强物价管理，建立价格监测、预警机制。继续加强统计巡查和城调、企调工作，搞好第二次全国农业普查。推进殡葬改革，巩固殡改成果。做好外事、口岸、气象、测绘、防灾减灾以及妇女儿童、老龄等工作。

　　八、着力扶持民生，建设和谐湛江

　　坚持为民办好实事。坚持为民务实，切实解决关系群众切身利益的问题。继续实施全民安居、扩大与促进就业、农民减负增收、教育扶贫、济困助残、外来员工合法权益保护、全民安康、治污保洁、农村饮水和城乡防灾减灾“十项民心工程”，务必取得新的成效，使经济社会发展成果更多地惠及普通百姓。

　　扩大就业和再就业。进一步落实“劳动者自主择业、市场调节就业和政府促进就业”的方针，强化就业工作目标责任考核，建立和完善覆盖城乡的公共就业服务体系。大力扶持就业困难人员创业，确保有就业愿望的新增城镇“零就业家庭”年内实现每户至少1人就业。落实税费减免、社保岗位补贴和小额担保贷款等优惠政策，努力扩大就业，全市新增就业岗位4万个。实施“农村青年培训工程”，年内转移农村劳动力就业16万人。稳步推进沿海渔民转产转业工作。

　　提高社会保障水平。继续完善社会保险市级统筹，扩大社会保险覆盖范围。加强地税部门全责征收社保费，确保养老保险基金收支平衡。实施大病救助和医疗补充保险办法，逐步解决困难企业、关闭破产转制企业职工的基本医疗保障问题。完善工伤、失业保险和企业职工生育保险制度。年内参加企业基本养老保险净增1.8万人，参加失业保险净增5000人，参加基本医疗保险净增1.2万人。

　　加强扶贫济困助残工作。突出抓好整村推进扶贫试点和产业化扶贫，支持扶贫龙头企业带动贫困户增收；培训农村贫困劳动力1.15万人，加快转移就业。巩固城乡最低生活保障制度，重点保障无经济来源、无劳动能力、家庭长期有老弱病残的对象和下岗退役伤残军人。推行城乡医疗救助制度，着力缓解农民因病致贫、因病返贫问题。新建村级敬老院120间。重视发展残疾人事业，进一步实施“扶残助学”工程，为残疾人倾注社会关爱。扩大法律援助，使更多困难群众得以维护自身的合法权益。

　　保障公共安全和社会稳定。切实保障食品安全、药品安全、生产安全、治安安全、环境安全，提高百姓生活的安全度。全面实施食品药品放心工程，加快食品安全和餐饮卫生检测体系、农产品检测中心和国家级海产品检测中心建设，建立市区肉菜、水产品市场准入制度，加强食品质量安全管理和检测工作，最大限度减少农药或有害物残留超标食品进市，让广大群众吃上放心的食品。强化各级安全生产责任制，突出抓好重点行业、重点领域的专项治理，及时排查和坚决消除重大安全隐患，加强消防基础建设，提高城乡消防能力，确保人民群众生命财产安全。高度重视信访工作，坚持领导定期接访、下访、重点巡访制度，完善社会利益协调和矛盾纠纷排查调处机制，及时化解各种社会矛盾；全面推进平安和谐社区建设；坚决打击交通、农产品流通等行业和地区非法垄断行为，维护市场经济秩序；完善社会稳定预警、公共突发事件应急处置机制和反恐、反走私工作体系，切实解决社会治安的突出问题，依法严厉打击各种违法犯罪和黑恶社会势力，全力维护社会稳定，保障人民群众安居乐业。

　　九、加强精神文明和民主法制建设，构建文明法治社会

　　深入推进精神文明建设。继续开展“爱国、守法、诚信、知礼”现代公民教育活动，引导全社会形成热爱祖国、遵纪守法、诚实守信、知书达礼的道德风尚和行为规范。广泛开展群众性精神文明创建活动，抓好文明社区、文明小区、文明行业和文明单位建设，提高市民的文明素质和城市的文明形象。

　　加强民主政治建设。自觉接受人大及其常委会的监督，认真执行人大及其常委会的各项决议、决定。积极支持人民政协履行政治协商、民主监督、参政议政职能。坚持市政府组成人员向市人大报告工作制度。重大决策实行社会公示和听证，认真听取各民主党派、工商联、无党派人士、各人民团体及社会各界的意见和建议。认真做好人大代表议案、建议和政协委员提案的办理工作。

　　继续推进依法行政。贯彻国务院《全面推进依法行政实施纲要》，落实各行政部门的职责分工，严格按照法定权限和程序行使权力、履行职责。深入推进相对集中行政处罚权和相对集中行政许可权工作。启动“五五”普法工作，规范管理律师、公证行业。

　　充分激发社会创造活力。大力推进各种形式的改革创新，进一步完善政策制度，通过健全激励机制、改革干部人事制度、建立扶贫脱困致富工程机制、实施“人才强市”战略和完善创业竞争机制，激发各级领导班子和干部队伍、人民群众、人才队伍以及市场主体的活力和创造精神，努力形成各尽所能、各得其所、和谐相处、奋发上进的局面。

　　十、加强政府建设，提高执政能力

　　面对改革开放和现代化建设的新形势、新任务和新要求，各级政府必须牢固树立“立党为公、执政为民”思想，充分体现党的先进性，不断加强自身建设，提高执政能力，努力建设人民满意的政府。

　　提高依法行政能力。加快建立权责明确、行为规范、监督有效、保障有力的行政执法体制，善于运用法律手段管理经济和社会事务，解决政府管理中的问题和矛盾，切实提高依法行政工作能力和水平。严格执行《广东省行政机关规范性文件管理规定》，把好规范性文件审查发布关，从源头上确保行政行为的合法性。完善行政复议制度，规范行政执法行为。

　　提高从严治政能力。梳理划清各级行政部门的权责范围，逐项列出向社会公布，切实解决行政部门职能交叉、权责不清、相互推诿的问题。加快完善决策责任、执行责任和追究责任制度体系，明晰权责关系，落实问责对象。全面推行行政执法责任制和评议考核制度，加强政府层级监督，加大对行政不作为、乱作为的问责力度，实行行政机关过错追究与个人过错追究相结合、一般工作人员责任追究与领导干部责任追究相结合，做到有权必有责、用权受监督、侵权要赔偿、违法要追究。全面实施《公务员法》，建立公务员绩效评价体系，把公务员管理和监督纳入法制化轨道，促进公务员勤政廉政、令行禁止、提高工作效能。

　　提高为民执政能力。强化为民服务意识，摒弃官僚作风，建设服务型政府、服务型机关。加强行政服务中心建设，进一步规范运行，提高效率。坚持推行一站式办公、一窗口服务，完善市长、局长办公日和部门办事工作日制度，大力推进政务公开，实行“阳光操作”，最大限度地方便企业和群众办事。切实转变机关作风，深入基层，了解民意，尽心竭力为群众谋利益，千方百计为群众排忧解难，使执政为民成为政府工作人员的行为规范和自觉行动。

　　提高科学理政能力。进一步处理好政府、市场、企业的关系，深入推进政资分开、政企分开、政事分开和政社分开，切实将政府职能转变到经济调节、市场监管、社会管理和公共服务上来，提高按市场经济规律履行职能的能力。推进政府决策的民主化科学化，完善公众参与、专家论证和政府决策相结合的行政决策机制，健全决策规则，规范决策程序，强化决策责任，提高政府科学决策水平。

　　提高廉洁从政能力。贯彻落实《建立健全教育、制度、监督并重的惩治和预防腐败体系实施纲要》和中纪委六次全会精神，全面落实党风廉政建设责任制，认真履行政府廉政承诺，严格执行廉洁从政的各项规定，做到清正廉洁，干净干事。要以改革统揽预防腐败工作，在更广范围发挥市场配置资源的基础性作用，减少滋生腐败的土壤。加强对重点领域、重点部门、重点资金和领导干部经济责任审计，继续实行审计结果和整改情况公告制度。严厉查处领导机关和领导干部中的以权谋私等违纪、违法行为，坚决纠正损害群众利益的不正之风，切实解决人民群众反映强烈的突出问题。

　　各位代表！湛江正处在加快发展的新时期，新的目标令人鼓舞，新的形势催人奋进，新的征程任重道远。让我们高举邓小平理论和“三个代表”重要思想伟大旗帜，牢固树立和认真落实科学发展观，在中共湛江市委的正确领导下，把握机遇，开拓创新，团结实干，发愤图强，为开创“十一五”时期湛江经济社会发展新局面，为加快建设现代化新兴港口工业城市和美丽的南方海滨城市而努力奋斗！
